# Elevation‐related climatic factors dominate soil free‐living nematode communities and their co‐occurrence patterns on Mt. Halla, South Korea

**DOI:** 10.1002/ece3.8454

**Published:** 2021-12-15

**Authors:** Zhi Yu, Shuqi Zou, Nan Li, Dorsaf Kerfahi, Changbae Lee, Jonathan Adams, Hyun Jeong Kwak, Jinsoo Kim, Sang‐Seob Lee, Ke Dong

**Affiliations:** ^1^ Department of Integrative Biotechnology Sungkyunkwan University Suwon South Korea; ^2^ Key Laboratory of Ministry of Education for Environment Change and Resources Use in Beibu Gulf Guangxi Key Laboratory of Earth Surface Processes and Intelligent Simulation Nanning Normal University Nanning China; ^3^ Department of Biological Sciences School of Natural Sciences Keimyung University Daegu Korea; ^4^ Department of Forestry, Environment and Systems Kookmin University Seoul South Korea; ^5^ School of Geographic and Oceanographic Sciences Nanjing University Nanjing China; ^6^ Department of Biological Sciences Kyonggi University Suwon‐si South Korea

**Keywords:** climate change, co‐occurrence network, mountain ecosystems, soil nematodes

## Abstract

Nematodes play vital roles in soil ecosystems. To understand how their communities and coexistence patterns change along the elevation as well as to determine the best explanatory factors underlying these changes, we investigated free‐living soil nematodes on Mt. Halla, South Korea, using an amplicon sequencing approach targeting the 18S rRNA gene. Our results showed that there was significant variation in the community diversity and composition of soil nematodes in relation to elevation. The network interactions between soil nematodes were more intensive at the lower elevations. Climatic variables were responsible explaining the elevational variation in community composition and co‐occurrence pattern of the nematode community. Our study indicated that climatic factors served as the critical environmental filter that influenced not only the community structure but also the potential associations of soil nematodes in the mountain ecosystem of Mt. Halla. These findings enhance the understanding of the community structure and co‐occurrence network patterns and mechanisms of soil nematode along elevation, and the response of soil nematodes to climate change on the vertical scale of mountain ecosystems.

## Graphical abstract

In this manuscript, we investigated the soil nematodes and their different feeding groups on Mt. Halla in South Korea. We found significant variation in the community diversity and composition of soil nematodes in relation to elevation. We also found the network between soil nematodes were more interacted at lower elevations on Mt. Halla. Climatic variables were more important than edaphic factors influencing the elevational zonation and the soil nematode co‐occurrence network.

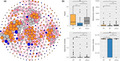

## 1 INTRODUCTION

Free‐living nematodes are the most abundant metazoans found in soil (Bongers, 1988). They are present in nearly all habitats on Earth and are especially ubiquitous in soil environments with their vital roles in soil food webs (Bongers, 1988). The response of nematode communities to various environmental disturbances (e.g., soil pH, temperature, soil humidity, nutrient level, and various metal ions) makes them suitable as indicators of different environments (Bongers & Ferris, 1999; Shao et al., 2017). Environmental variables are strong filtering effects on nematode communities in terms of both climatic and edaphic factors (da Silva et al., 2020; Siebert et al., 2019; Thakur et al., 2017, 2019). It has been shown that changes in the climatic variables (e.g., temperature and precipitation) directly affect the living environment and consequently influence the community diversity and structure of soil nematodes (da Silva et al., 2020; Nielsen et al., 2014; Song et al., 2017; Thakur et al., 2017). For example, a temperature that is too high or too low inhibits the growth and activity of soil nematodes because they have specific optimal temperatures for growth and propagation (Bird & Wallace, 1965); excessive precipitation or drought can affect the balance between water and air in the nematode living space (Neher, 2010). On the other hand, although it has been suggested that soil nematodes may not be sensitive to soil heterogeneity at local scales, heterogeneity in soil properties (e.g., soil pH, soil moisture, and nutrient levels) also works as an important driving force for a broad range of belowground communities, including soil nematodes (Hoschitz & Kaufmann, 2004; Quist et al., 2019; Reid et al., 2019). Many previous studies have indicated the important role of soil properties, such as organic nutrient deposition, soil pH, and soil moisture, in affecting communities of soil nematodes in different ecosystems (Kitagami et al., 2017; Sun et al., 2013). However, although both climatic and edaphic variables have important influences on nematode communities (Li et al., 2020; Namu et al., 2018; Siebert et al., 2020; St‐Marseille et al., 2019), it is not clear which sets of factors contribute most strong to variation in free‐living soil nematode communities in mountain environments (Dong et al., 2017a; Nielsen et al., 2014).

Mountain environments provide ideal conditions for studying variation in communities and determining the fundamental environmental variables of the community variation as both the climatic and edaphic conditions significantly change within a short distance along elevations (Hodkinson, 2005). It has been suggested that elevation strongly influence soil nematodes in strengthening ecological specialization along elevation‐related environmental gradients (Dong et al., 2017a; Kergunteuil et al., 2016), and the harsh environments at higher elevations are known to drive compositional modifications in the nematode community based on ecological traits, thus conferring local adaptations (Kergunteuil et al., 2016). Many studies have confirmed the significant elevational zonation of soil free‐living nematode community due to varying environmental conditions along the elevational gradient although it was not always the same (Dong et al., 2017a; Kergunteuil et al., 2016).

Co‐occurrence network analysis gives a way to explore the influence of environmental variables on the complexity and stability of the network (Barberán et al., 2012). It has been increasingly used in community ecology to reflect the associations between taxa in the ecosystem, since the positive or negative interactions effectively reflect patterns of co‐existence or mutual exclusion between pairs of taxa (Yuan et al., 2021). Even though many previous studies indicated that different environmental variables significantly affect the complexity and stability of the co‐occurrence network of microorganisms, to the best of our knowledge, little is known about how soil free‐living nematodes co‐occur, as well as how their network co‐occurrences vary in relation to elevation and environmental variables (Cardinale et al., 2015; Fan, Weisenhorn, Gilbert, Shi, et al., 2018; Wang et al., 2018; Yuan et al., 2021).

Here, we conducted our study on Mt. Halla, which is the highest mountain in South Korea, to investigate how the soil free‐living nematode communities change along the elevation, and which set of the environmental variables is the most effective explanatory factors underlying these changes. We hypothesized (1) the community diversity decreases with increasing elevation as the environmental conditions become more physiologically extreme and less productive at higher elevations, as has been suggested by previous studies (Andriuzzi et al., 2018; Liu et al., 2019). (2) The network interactions among soil nematodes will be more complex at lower elevations, where the temperature is higher. This is consistent with the common paradigm in ecology that an increase in temperature promotes biological activity rates of species, in turn facilitating the interaction between the species (Yuan et al., 2021). (3) Climatic variables have a more important effect in determining nematode communities compared to edaphic variables in term of community diversity, community structure and community co‐occurrences, since along elevation gradients climates usually become more distinctive than soil characteristics (Nisa et al., 2021).

## 2 MATERIALS AND METHODS

### 2.1 Site description and soil sampling

Mt. Halla is a volcanic mountain located in Jeju Island, South Korea. It has a wide range of climatic conditions with a subtropical maritime climate in low‐elevation areas and gradually changes to a subalpine climate as the elevation increases (Ahn & Yun, 2020). The mean annual temperature (MAT) is 14.7°C, with minimum temperatures in January of 5.1°C and maximum in August of 25. 4°C. The MAT at the top of Mt. Halla is 3.7°C, while it is much colder in winter (Lee et al., 1999; Won et al., 2006). The mean annual precipitation (MAP) ranges from 1130 to 2200 mm in the lower elevation sites, 1960 to 2340 mm in the middle, and 3190 to 5870 mm in the higher elevation sites (Ahn & Yun, 2020). Analysis of precipitation data for the 10 years from 2003 to 2012 shows the MAP of Mt. Halla proportional to the elevation (Choi, 2013).

A total of 30 samples were collected at six elevations along elevational isoclines separated by ~300 m of elevation in September, 2019. At each elevation, the five samples separated at least 20 m. Within each individual sample, five soil cores were combined using the top‐soil (0–10 cm depth directly below the litter layer) from four corners and central point of the 1 m × 1 m quadrat of each sample to make a composite sample. Nematodes were collected using a modified Baermann funnel method following Dong et al. (2017a).

### 2.2 Classification of soil free‐living nematodes

Nematode DNA was extracted from the centrifuged nematode pellet using the DNeasy PowerSoil Kit (Germany) following manufacturer's instructions. The isolated DNA was stored at −80°C until the PCR stage. NF1/18Sr2b primer sets (NF1, 5’‐GGTGGTGCATGGCCGTTCTTAGTT‐3’, 18Sr2b, 5’‐TACAAAGGGCAGGGACGTAAT‐3’) were used to amplify the 18S rRNA genes (Du et al., 2020; Fan et al., 2020). DNA library preparation and sequencing were done on Illumina MiSeq sequencing platform in Macrogen, Inc., Seoul, Korea. The sequence data were processed using mothur following the Miseq SOP. Briefly, the forward and reverse direction files were combined using the make.contiq command. Sequences with any ambiguous bases and with lengths <300 bp and lager than 450 bp were removed using the screen.seqs command. Chimeric sequences were detected and removed via the Chimera Vsearch algorithm in de novo mode contained within Mothur. Rare sequences (less than 10 reads) were removed to avoid the risk of including spurious reads generated by sequencing errors. High‐quality sequences were assigned to operational taxonomic units (OTUs) at ≥97% similarity level and classified against SILVA database (http://www.arb‐silva.de/) (Dong et al., 2017b). The feeding groups of the OTUs were determined according to Yeates (Yeates et al., 1993) and NEMAGuild (Nguyen et al., 2016). Four feeding groups were created: bacteria‐feeding nematodes (BF), fungi‐feeding nematodes (FF), plant‐feeding nematodes (PF), and omnivore/predator (OP). Since the proportions of FF and PF nematodes were too small, they were not included in the analyses.

### 2.3 Soil physicochemical analysis

The soil physical and chemical properties of each sample were analyzed in the National Instrumentation Center for Environmental Management, Seoul National University. The parameters analyzed were pH, total organic carbon (TOC), total nitrogen (TN), ammonium (${\text{NH}}_{4}^{+}$), nitrate (${\text{NO}}_{3}^{‐}$), available phosphate (P_2_O_5_), moisture, and soil texture (being indicated by the proportion of sand and the proportion of silt and clay). MAT and MAP data of each site were obtained from national digital climate maps constructed by the National Center of AgroMeteorology at the Korean Meteorological Administration (Chun & Lee, 2018; Yun, 2010).

### 2.4 Statistical analysis

Nematode species richness was calculated and used in the analysis on the effect of environmental variables. To test the influences of elevation on nematode species richness, the linear or quadratic model was selected based on the lower value of Akaike's information criterion (AIC). Community compositional dissimilarities were estimated based on the Bray–Curtis distance of species abundance transformed by “decostand” function (Hellinger method) in the “vegan” package in R (Oksanen et al., 2015). For the relationship between community dissimilarity and elevation, principal coordinates analysis (PCoA) and permutational multivariate analysis of variance (PERMANOVA) were performed in R package “vegan” (Oksanen et al., 2015).

We classified the measured environmental variables to two categories: climate variables (MAT and MAP) and edaphic variables (pH, TOC, TN, ${\text{NH}}_{4}^{+}$, ${\text{NO}}_{3}^{‐}$, P_2_O_5,_ moisture, and soil texture). To test the relative importance of environmental variables in driving nematode species richness, we used random forest analysis in the “randomForest” in R package (Liaw & Wiener, 2002). The importance of variables was determined by the value of %IncMSE (increased in mean squared error) calculated by the “importance” function.

To understand which nematode families mostly contribute to community dissimilarity between the elevational transects, SIMPER analysis was conducted to identify the contribution of nematode families‐level differences to the overall community difference and the contribution of nematode families was illustrated in boxplots. Multiple regression models were used to test the impact of environmental factors on the major nematode families. Mantel tests were used to test the correlations between environmental variables and community composition. The Bray–Curtis distance matrices for species abundance and Euclidean distance matrices for environmental factors standardized with “decostand” function of the “vegan” package were used in Mantel test. Mantel tests and SIMPER analysis were performed in R package “vegan” (Oksanen et al., 2015).

All the above analyses were simultaneously conducted on OTU‐level, and the results exhibited identical patterns (data not shown).

### 2.5 Network construction and topological feature analysis

The co‐occurrence network of nematode communities were constructed based on the Spearman correlation matrix with the “WGCNA” package in R (Ma et al., 2016). OTUs with relative abundance more than 0.01% of the total nematode sequences were considered (Ma et al., 2016). *p*‐values for multiple testing were adjusted using the Benjamini and Hochberg false discovery rate (FDR) control procedure in R package “multtest” (Benjamini et al., 2006). Correlation coefficients (*ρ*) with statistically significance (*p* < .01) and an absolute value over 0.60 were retained in the network analyzes. The nodes represent the main OTU, and the edges correspond to the correlation links between nodes.

The network topology features were calculated using the “igraph” package in R. Subnetworks were constructed for each soil sample by “subgraph” function in the “igraph” package (Csardi & Nepusz, 2006). The topological characteristics of each node in the network and the network‐level topological characteristics of each sample were used to describe the topological characteristics of the nematode co‐occurrence network following Chen et al. (2021). Four node‐level network topology features (degree, betweenness, closeness, and eigenvector centrality) and thirteen network‐level topology features (nodes number, edges number, average degree, average path length, edge density, diameter, modularity, global clustering coefficient, degree centralization, betweenness centralization, eigenvector centralization, closeness centralization, and mean connectivity) were used (Table S1). Gephi platform was used to visualize the nematode co‐occurrence network (http://gephi.github.io/). Mantel tests were used to test the correlations between environmental variables and network topology features in R “vegan” package (Oksanen et al., 2015). Linear or quadratic model selection was based on Akaike's information criterion (AIC). Multiple regression model with variance decomposition analysis was used to test the influence of each environmental variable on the network topology characteristics in R with “relaimpo” package (Grömping, 2007).

### 2.6 Keystone taxa analysis

In this study, the within‐module connectivity (*z*‐score) and among‐module connectivity (c‐score) of each node were calculated based on methods of metabolic networks with the “sqldf” package in R (Guimera & Amaral, 2005). In order to better describe the role of the hubs, based on their within‐module degree (*z*‐score) and participation coefficient (*c*‐score) threshold value, network hubs (*z*‐score >2.5; *c*‐score >0.6), module hubs (*z*‐score >2.5; *c*‐score <0.6), connectors (*z*‐score <2.5; *c*‐score >0.6), and peripherals (*z*‐score <2.5; *c*‐score <0.6) were defined of each nodes (Fan, Weisenhorn, Gilbert, & Chu, 2018; Poudel et al., 2016). The network hubs were highly connected both within and between modules, the module hubs were highly connected within a module, the connectors were highly connected between modules and provided links among multiple modules, and the peripherals mean the nodes had few or no links to other nodes (Poudel et al., 2016). Network hubs, module hubs, and connectors were termed keystone network topological features; these are considered to play important roles in maintaining community stability and responding to environmental stress (Tylianakis & Morris, 2017; Yang et al., 2021). Thus, we define the OTUs associated with these nodes as keystone species. Based on the abundance data at the species level, multiple regression models were used to assess the correlation between the abundance of keystone species and changes in environmental factors in R with “relaimpo” package (Grömping, 2007).

## 3 RESULTS

### 3.1 Community features of the soil free‐living nematodes

**FIGURE 1:**
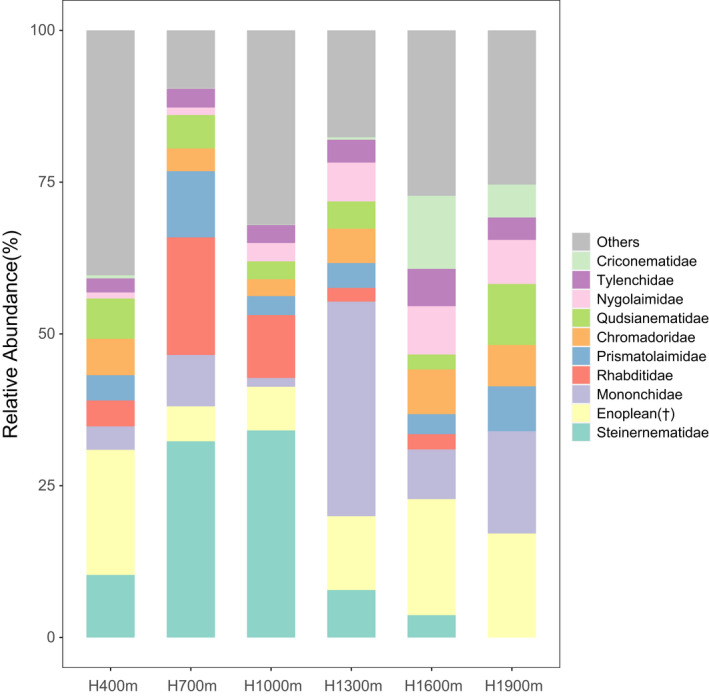
Relative abundance (%) of nematode families on different elevational isoclines. Enoplean(†): unclassified Enoplean family

**FIGURE 2:**
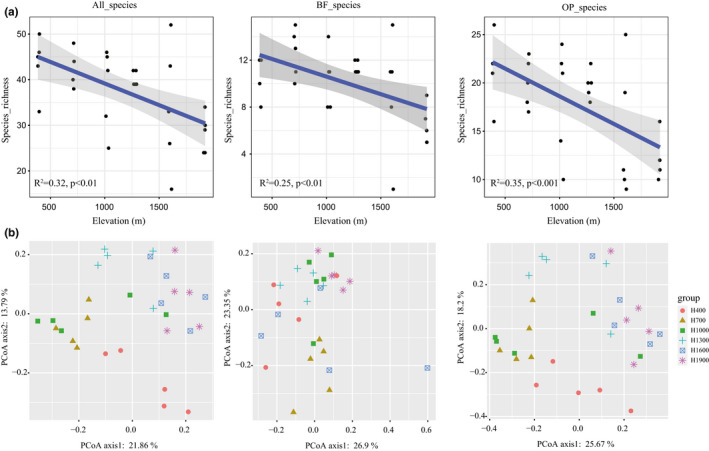
The monotonically decreasing patterns of the nematode species richness along elevation (a); PCoA plots of trends in the composition of nematode community based on Bray–Curtis dissimilarity in Mt. Halla (b)

The high‐quality sequences were assigned to 128 genera from 39 families. Steinernematidae was the most dominant family, taking 14.7% of the reads, the second dominant family was an unclassified Enoplean family taking 13.7% of the reads, followed by Mononchidae (12.4%), Rhabditidae (6.5%), Prismatolaimidae (5.5%), Chromadoridae (5.4%), Qudsianematidae (5.4%), Nygolaimidae (4.5%), Tylenchidae (3.7%), and Criconematidae (3.1%) (Figure 1). These families together covered near 80% of the total nematode reads. The relative abundance of Steinernematidae, Nygolaimidae and the unclassified Enoplean family were significantly correlated with elevation (*p* < .05, Figure S1). At the genus level, in addition to the unclassified genus, *Steinernema_glaseri* was the most dominant genus, taking 13.6% of the reads, followed by *Achromadora_sp*.*_JH*‐*2004* (4.2%), *Prismatolaimus_cf*.*_dolichurus_JH*‐*2004* (3.3%), *Rhabditis_cf*.*_terricola_JH*‐*2004* (3.2%) (Figure S2). The nematode richness exhibited a significant correlation with elevation, monotonically decreasing along the gradient (*R* ^2^ = 0.32, *p* < .01). Similarly, the richness of different feeding groups, that is, BF nematodes and OP nematodes, also exhibited monotonically decreasing patterns along elevation (*R* ^2^ = 0.25, *p* < .01; *R* ^2^ = .35, *p* < .001, respectively; Figure 2a). The community structure of the soil nematodes was highly variable across the soils represented by different elevations according to PCoA plots based on the Bray–Curtis distance (PERMANOVA, *p* < .001; Figure 2b, Table S2). The results of SIMPER analysis suggested the dominant families, such as Steinernematidae, Mononchidae, and Rhabditidae, made a greater contribution than other families to the community structure difference between elevational transects, and Steinernematidae was the most important contributor to the community dissimilarity (Figure S3). The analyses on community diversity and community structure were simultaneously conducted on OTU‐level, and the results exhibited identical patterns (data not shown).

### 3.2 Co‐occurrence patterns of the soil nematode communities

**FIGURE 3:**
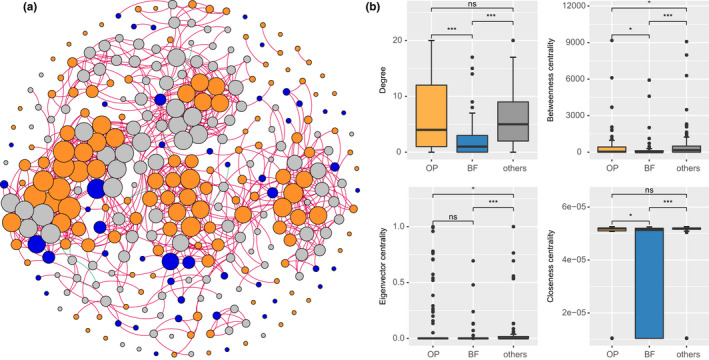
Co‐occurrence network of soil nematodes on Mt. Halla. Coloring is based on different nematode‐feeding groups (yellow: OP; blue: BF; gray: others). The red edge represents a positive correlation, and the green edge represents a negative correlation (a). The node‐level topological features of different feeding groups, the degree, betweenness, closeness, and eigenvector centrality are shown. *p < .05, **p < .01, and ***p < .001 (b)

The co‐occurrence network of the nematodes was established based on Spearman correlation of 1013 high‐quality operational taxonomic units (OTUs). Overall, there was 311 nodes and 930 edges, with 99.03% edges were positive (Figure 3a; Table S3). 136 and 49 among the total nodes belong to OP nematodes and BF nematodes, respectively. The Unclassified Enoplean family (19.94%), Chromadoridae (8.04%), Tylenchidae (6.75%), and Steinernematidae (6.11%) played the most important roles in the network. The node‐level topological feature values (i.e., degree, betweenness, and closeness centrality) between OP nematodes were significantly higher than BF nematodes (*p* < .05), suggesting OP nematodes contributed a greater proportion to the nematode network than BF nematodes (Figure 3b). Interestingly, the significant negative linear relationship between elevation and the number of edges, average degree, and the number of nodes, and the significant linear positive relationship between elevation and modularity indicated that the network connection between soil nematodes were strengthened with the decrease of elevation (Figure S4). This also indicated that the network interactions between soil nematodes were more intensive at the lower elevations.

### 3.3 Climatic factors dominating the soil nematode community

**FIGURE 4:**
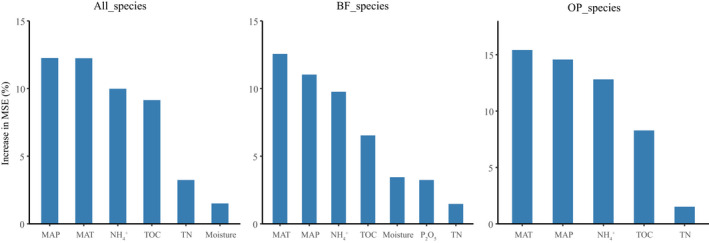
The relative importance of climate and edaphic factors for nematode species richness on Mt. Halla

**FIGURE 5:**
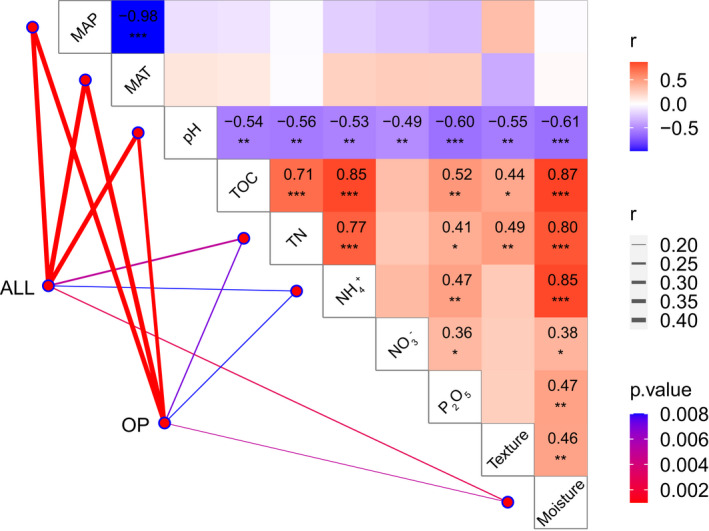
The Mantel test of nematode communities and environmental factors and the correlation analysis between environmental factors on Mt. Halla. The climatic factors include mean annual precipitation (MAP), mean annual temperature (MAT); the edaphic factors include soil pH (pH), total organic carbon (TOC), total nitrogen (TN), ammonium (${\text{NH}}_{4}^{+}$), nitrate (${\text{NO}}_{3}^{‐}$), available phosphate (P_2_O_5_), soil moisture (Moisture), and soil texture (Texture). *p <  .05, **p <  .01, and ***p <  .001

Random forest analysis was applied to determine the main environmental factors that dominate the nematode diversity. It is suggested that MAT and MAP, both of which are climatic variables, were the most important factors affecting the species richness of the free‐living nematode community, as well as the BF and the OP nematodes (Figure 4). The Mantel test indicated that MAT was the most important factor influencing the soil nematode community structure across elevation on Mt. Halla (*R* = 0.44, *p* < .001), followed by MAP, soil pH, and TN (Figure 5; Table S4). Multiple regression models were used to assess the influences of environmental variables on the ten most abundant families. The results showed MAT and MAP, again, were consistently important in most of the families (8 out of 10) (Figure S5). Also, climatic factors were the main environmental factors affecting the topological features of soil nematode networks suggested by both the mantel test and the multiple regression model. While MAP and MAT contributed the largest explanation on network topology features (*R* = 0.25, *p* < .01; *R* = 0.21, *p* < .01) (Table S5), the multiple regression model showed that the climatic factors explained a larger proportion than edaphic factors on the variation of the topological features in eight main network topological features (Figure S6).

### 3.4 Identification of keystone taxa and association with environmental factors

**FIGURE 6:**
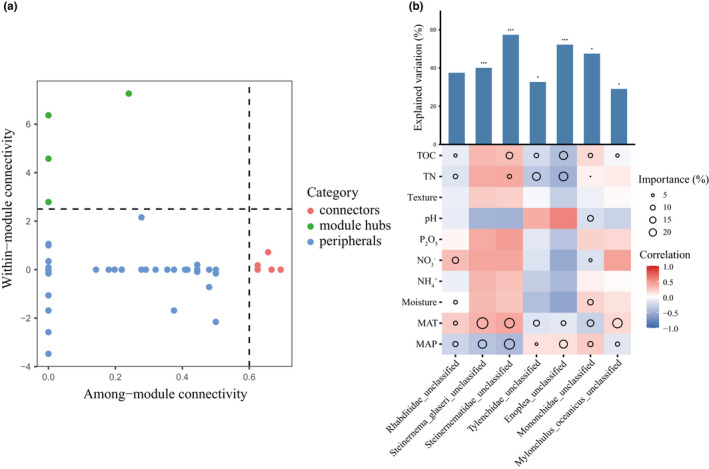
Identification of potential keystone species and their abundance in relation to environmental factors. (a) Z‐C plot showing the classification of nodes to identify putative keystone species of ecological network. Each symbol represents an OTU. (b) The correlation between environmental factors and the abundance of keystone species along with the best multiple regression model. Circle size represents the variable's importance. Colors represent Spearman correlations. The climatic factors include mean annual precipitation (MAP), mean annual temperature (MAT); the edaphic factors include soil pH (pH), total organic carbon (TOC), total nitrogen (TN), ammonium (${\text{NH}}_{4}^{+}$), nitrate (${\text{NO}}_{3}^{‐}$), available phosphate (P_2_O_5_), soil moisture (Moisture), and soil texture (Texture). *p <  .05, **p < 0.01, and ***p <  .001

In the soil nematode co‐occurrence network, peripheral nodes accounted for 93.88% of the total number of nodes, connectors accounted for 4.08%, module hubs accounted for 2.04%, and there were no network hubs (Figure 6a). The 4 modular hub nodes all belong to the Steinernematidae family, and the 7 connector nodes belong to the Rhabditidae, Mononchidae, Enoplea_unclassified, and Tylenchidae families. Based on the abundance data at the species level, multiple regression models were used to assess the correlation between the abundance of keystone species and changes in environmental factors. We found that MAP and MAT were the two variables most closely related to the abundance of keystone species, while TOC and TN also play an important role (Figure 6b).

## 4 DISCUSSION

### 4.1 Community of the soil free‐living nematodes

The elevational trends of species diversity have been well studied, and most studies have shown that species diversity decreases with increasing elevation (Gaston, 2000; McCain, 2005; Rahbek, 2005; Zhi‐Yao & Jing‐Yun, 2004). In this study, we found that soil nematode diversity significantly decreased with the increasing elevation on Mt. Halla, with a higher nematode richness found at lower elevations. This monotonically decreasing trend in diversity along the elevation is consistent with previous studies on soil nematodes and soil fauna (Andriuzzi et al., 2018; Liu et al., 2019; Smith‐Ramírez et al., 2007). However, different types of elevational diversity patterns of soil nematodes have also been reported (Kergunteuil et al. (2016). For example, Kergunteuil et al. (2016) found that soil nematode diversity was highest in alpine grasslands above 3000 m in the Alps, probably because of the diverse vegetation, rich organic soil layers, and abundant rainfall above 3000 m. On the other hand, Dong et al. (2017) found that the soil nematode diversity showed a mid‐elevation maximum on Mt. Norikura, Japan, the highest annual precipitation at 2000–2500 m on Mt. Fuji, which was deemed responsible for the diversity maximum. Regardless of the type of diversity pattern along elevation, annual precipitation seems to consistently play a very important role in influencing the diversity and abundance of soil nematodes.

In the present study, the community structure of the soil nematodes was found to be highly variable across the soil samples collected at different elevations, which is consistent with previous studies showing that nematode community structure varies significantly with the elevation (Kergunteuil et al., 2016; Liu et al., 2019; Tong et al., 2010). The soil nematode communities at the high‐elevation areas were characterized by the presence of Mononchidae, Nygolaimidae, Criconematidae, and an Enoplean family, while Steinernematidae, Mononchidae, and Rhabditidae made a greater contribution to the community structure differences between the elevational transects. Mononchidae, which is adapted to extreme cold temperatures (Dong et al., 2017a; Keshari et al., 2018), is a ubiquitous component and an important contributor to belowground soil communities in soils at high elevation. This is consistent with the previous profiling of soil nematodes in the high‐elevation region of Mt. Norikura, Japan (Dong et al., 2017a, 2017b). The relative abundance of the Enoplean family was high; however, it made a relatively small contribution to the variation in communities as compared to other predominating families such as Mononchidae and Rhabditidae, indicating that the high relative abundances of certain families do not result in a correspondingly large contribution to the nematode community dissimilarity between the elevational transects. Steinernematidae, which represents a group of soil nematodes parasitizing various insects, is sensitive to changes in temperature, and precipitation in soil (Rohde et al., 2010). In this study, Steinernematidae was observed to be the most important contributor to the community dissimilarity between elevations; it was more abundant at the mid‐elevation levels and was absent at the high elevation on Mt. Halla. Given that the elevation‐related climatic variables, MAT and MAP, were found to be significantly correlated with the relative abundance of Steinernematidae, and that the highest abundance of Steinernematidae was found at the intermediate level of MAT and MAP, it is plausible to conclude that climatic variables are of great importance in shaping the soil nematode communities (Figure S7).

### 4.2 Climatic factors dominate the soil free‐living nematodes

Temperature has been considered an important climatic factor shaping soil nematode communities because of the strong filtering effects that influence the metabolism of nematodes with different temperature adaptabilities (Bai et al., 2010; Schuur et al., 2007). Moreover, temperature regulates the decomposition rate of soil organic matter, and higher temperatures usually accelerate the conversion of available soil nutrients (Schuur et al., 2007; Song et al., 2017). In this study, MAT indeed was observed to significantly influence the entire nematode communities as well as the omnivorous feeding group; however, there was no environmental variables significantly correlated to the community composition of bacteria‐feeding nematodes. Compared to the other environmental variables, soil pH was found to be a more important explanatory variable underlying the community composition of the bacteria‐feeding nematodes. This could be explained by the direct feeding relationships between BF nematodes and bacteria, whose abundance and diversity are greatly influenced by soil pH (Nottingham et al., 2018; Shen et al., 2015, 2020; Wang et al., 2015). Interestingly, previous studies have suggested that the bacterial communities on Mt. Halla are predominantly influenced by climatic variables, such as MAT and MAP (Singh et al., 2014); hence, there is a need for further research to reveal the complex relationship between soil nematodes and bacteria on Mt. Halla.

Precipitation is another important climatic factor that shapes the soil nematode communities by not only directly affecting the movement, feeding, growth, and reproduction of individual soil nematodes, but also indirectly affecting the community by changing the net primary productivity of local vegetation, organic nutrient, mineral levels, and activities of the surrounding microorganisms (Bai et al., 2008; Kardol et al., 2011; Wang et al., 2006). Greater precipitation may be expected to provide moister; however, aerated soils still are physiologically favorable for nematodes and their feeding activity, thus resulting into higher diversity and abundance of soil nematodes. Nonetheless, both the lowest annual precipitation and the highest community diversity were observed at the low‐elevation areas on Mt. Halla in this study. Although soil nematode communities have higher diversity and abundance in more humid environments (Landesman et al., 2011; Ruan et al., 2012), it is believed that too much precipitation will in turn greatly reduce the diversity of soil nematodes; the largest diversity and abundance are expected to occur at the intermediate level of precipitation (Song et al., 2017). Even though many factors may account for this, it is more likely that soil nematodes are more restricted due to the decline in oxygen levels caused by excessive soil moisture (Van Gundy & Stolzy, 1961).

### 4.3 Climatic factors affect soil nematode co‐occurrence patterns

Previous studies have shown that environmental factors significantly affect the co‐occurrence patterns of soil microorganisms (Chen et al., 2021; Yuan et al., 2021). For example, the network interaction between bacteria and archaea was enhanced with increasing soil pH increased in the alpine grasslands of the Tibetan Plateau (Chen et al., 2021), and an increase in temperature enhanced the complexity and stability of the soil microbial network of the tallgrass prairie ecosystem (Yuan et al., 2021). However, ours is the first study to reveal the importance of environmental variables, especially climatic factors, in the co‐occurrence patterns of soil nematode communities in mountain environments. As expected, the nematode co‐occurrence networks on Mt. Halla were more connected in the low‐elevation areas, which has lower MAP and higher MAT. Yang et al. (2021) suggested that climatic factors may regulate the co‐occurrence patterns indirectly through soil characteristics. However, the present study did not find strong influences of the climatic variables on the edaphic variables. Interestingly, positively correlated edges accounted for more than 99% of the soil nematode co‐occurrence networks in this study. Edges of co‐occurrence network reflect the interactions between the species involved in the network. While negatively correlated edges are able to reflect competitive behaviour between species, positively correlated edges mainly represent mutual symbiosis, cross‐feeding, and other cooperative behaviors between the species (Ahn & Yun, 2020). Our results suggest that the soil nematode population on Mt. Halla lives in environments with relatively rich resources and mutually beneficial symbiotic association. Our results on node‐level topological values of the soil nematodes suggested that OP nematodes were more important than BF nematodes and that other feeding types (i.e., plant‐feeding and fungi‐feeding) also played important roles in the network. However, the contribution of plant‐feeding nematodes and fungi‐feeding nematodes to the communities was not analyzed and described in this study because their proportions were relatively small compared to those of OP and BF. Therefore, the role of PF and FF nematodes in the entire nematode community co‐occurrence network requires further in‐depth analysis.

## 5 CONCLUSION

This study provides an understanding of the ecology of free‐living soil nematodes in a mountain ecosystem. The nematode communities on Mt. Halla exhibited strong elevational zonation which was mainly driven by elevation‐related climatic factors. These climatic factors were also shown to have a significant influence on the co‐occurrence network patterns of the free‐living soil nematodes. With the trend of global warming in recent years, it is of great importance to understand how the nematode community will respond to such climate change, and our results demonstrating how the nematode communities respond to elevation‐related climatic changes provide theoretical framework for future studies on the response of soil nematode communities to climate change.

## CONFLICT OF INTEREST

The authors declare no conflict of interest.

## AUTHOR CONTRIBUTIONS

**Zhi Yu:** Conceptualization (lead); Investigation (lead); Methodology (lead); Software (lead); Visualization (lead); Writing – original draft (lead); Writing – review & editing (lead). **Shuqi Zou:** Conceptualization (supporting); Investigation (lead); Methodology (supporting); Software (supporting); Visualization (supporting); Writing – review & editing (supporting). **Nan Li:** Conceptualization (supporting); Funding acquisition (equal); Methodology (supporting); Writing – review & editing (supporting). **Dorsaf Kerfahi:** Formal analysis (supporting); Methodology (supporting); Writing – review & editing (supporting). **Changbae Lee:** Conceptualization (supporting); Methodology (supporting); Software (supporting); Writing – review & editing (supporting). **Jonathan Adams:** Conceptualization (supporting); Methodology (supporting); Writing – review & editing (equal). **Hyun Jeong Kwak:** Methodology (supporting); Software (supporting). **Jinsoo Kim:** Methodology (supporting); Software (supporting). **Sang‐Seob Lee:** Conceptualization (supporting); Investigation (supporting); Resources (supporting); Writing – review & editing (supporting). **Ke Dong:** Conceptualization (lead); Data curation (lead); Formal analysis (equal); Investigation (lead); Methodology (equal); Project administration (lead); Resources (equal); Software (equal); Supervision (lead); Validation (lead); Writing – original draft (supporting); Writing – review & editing (lead).

## Supporting information

Fig S1

Fig S2

Fig S3

Fig S4

Fig S5

Fig S6

Fig S7

Appendix S1

## Data Availability

The datasets generated from this study on Mt. Halla are deposited in NCBI Sequence Read Archive (SRA) under BioProject number PRJNA691425. The datasets of sampling locations, classification of reads, classification of OTUs, OTUs and environmental variables tables are available as Appendix S1 and as a Dryad repository, https://doi.org/10.5061/dryad.wpzgmsbp0.
