# Supplementary figures and images for: Elevation‐related climatic factors dominate soil free‐living nematode communities and their co‐occurrence patterns on Mt. Halla, South Korea
Source: Ecol Evol. 2021 Dec 15;11(24):18540–51. doi: 10.1002/ece3.8454 (PMC8717350; doi:10.1002/ece3.8454)

Steinernematidae

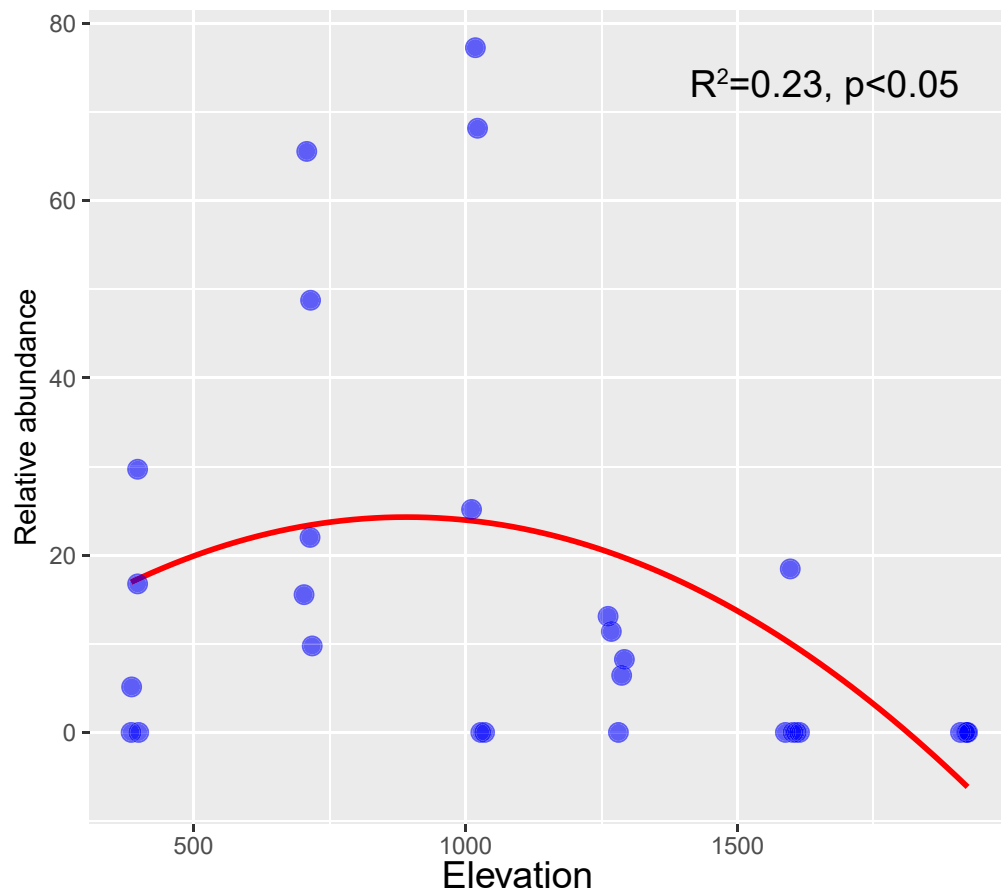

Nygolaimidae

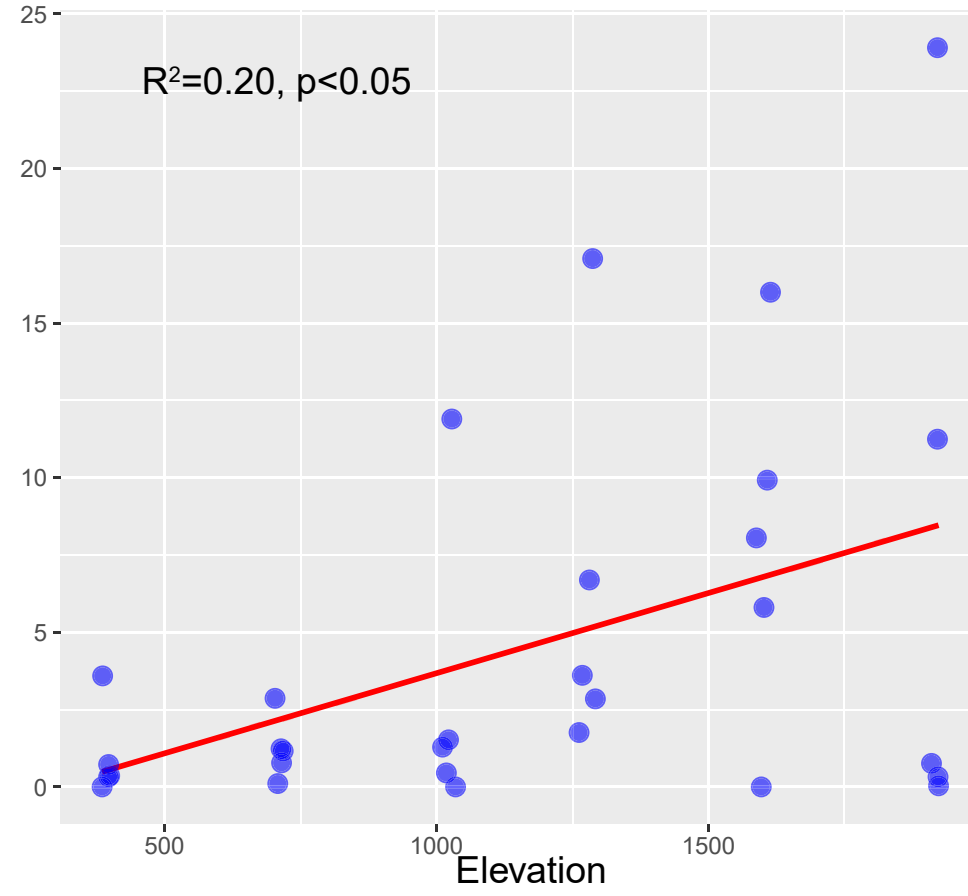

Unclassified.Enoplean.family

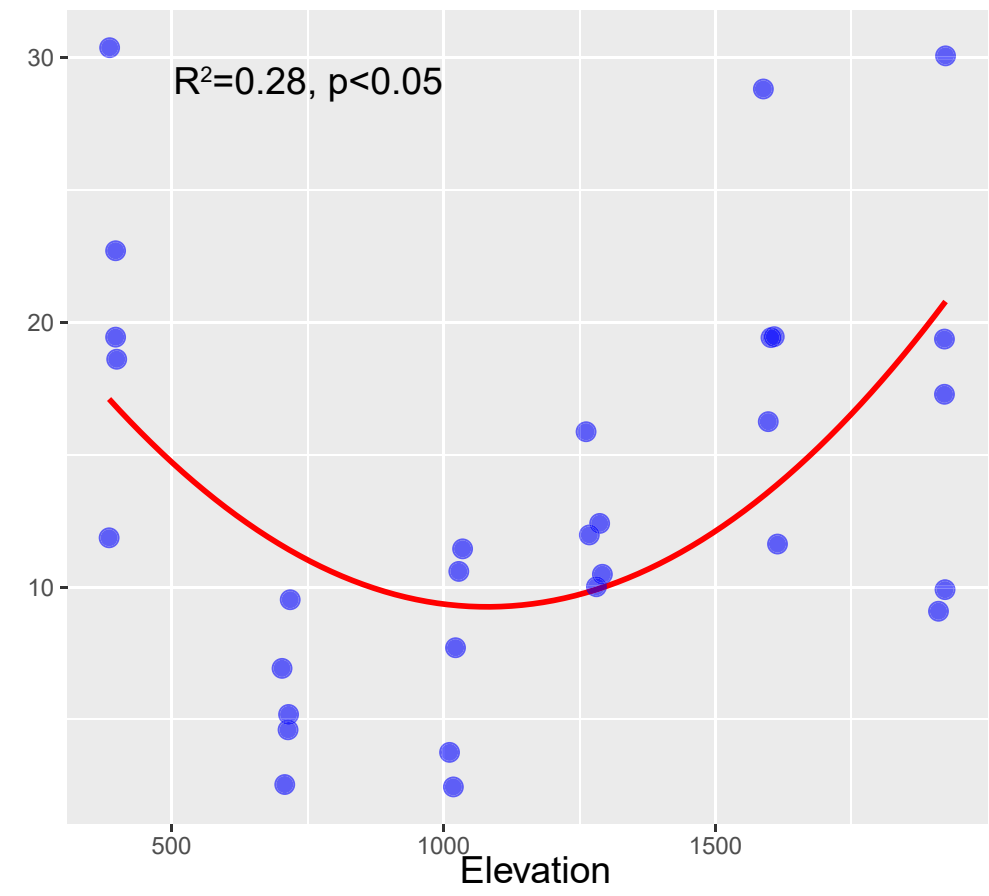

Supplement: Supplementary file 1 — Fig S1 [file ECE3-11-18540-s005.pdf]

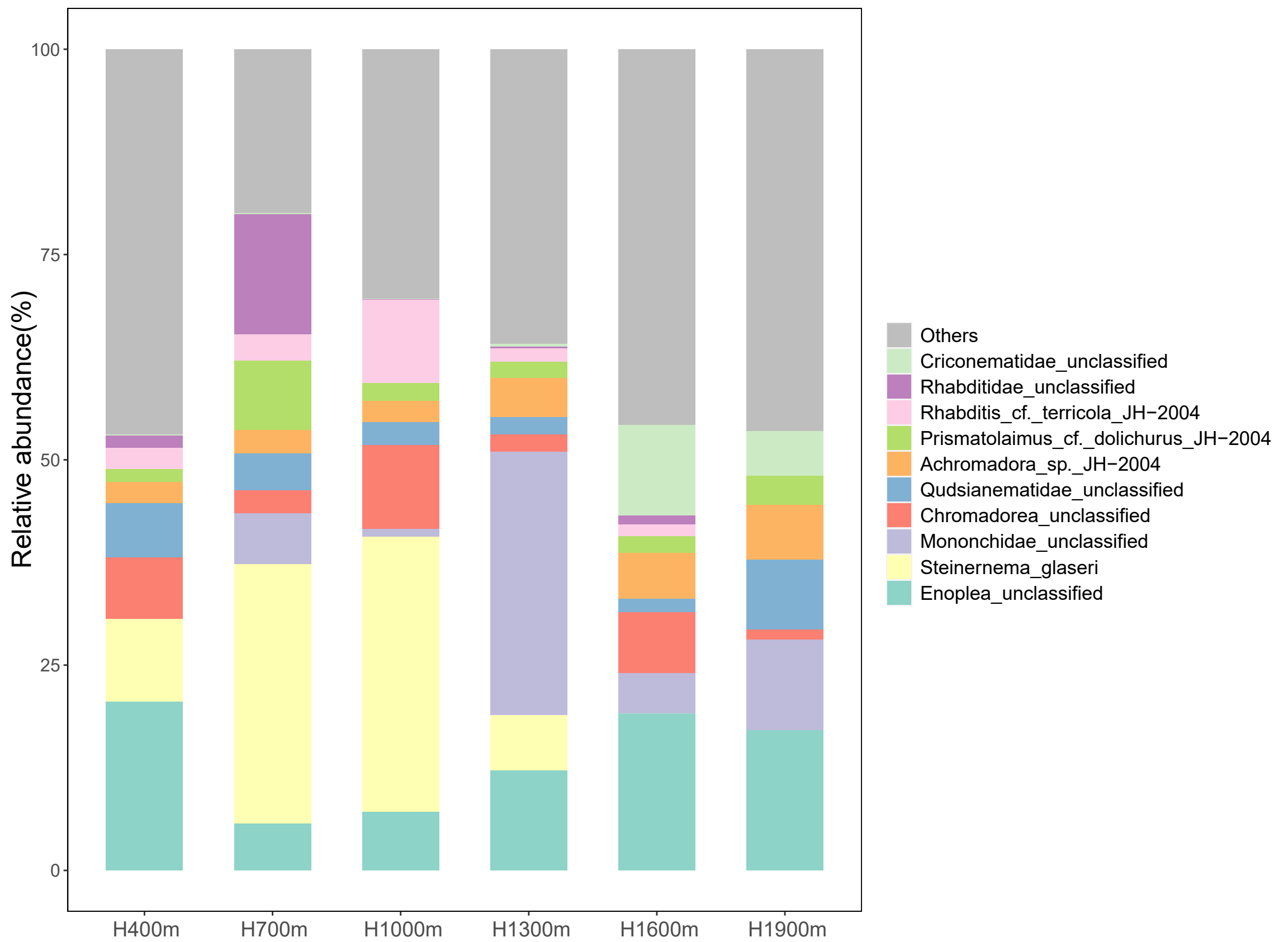

Supplement: Supplementary file 2 — Fig S2 [file ECE3-11-18540-s008.pdf]

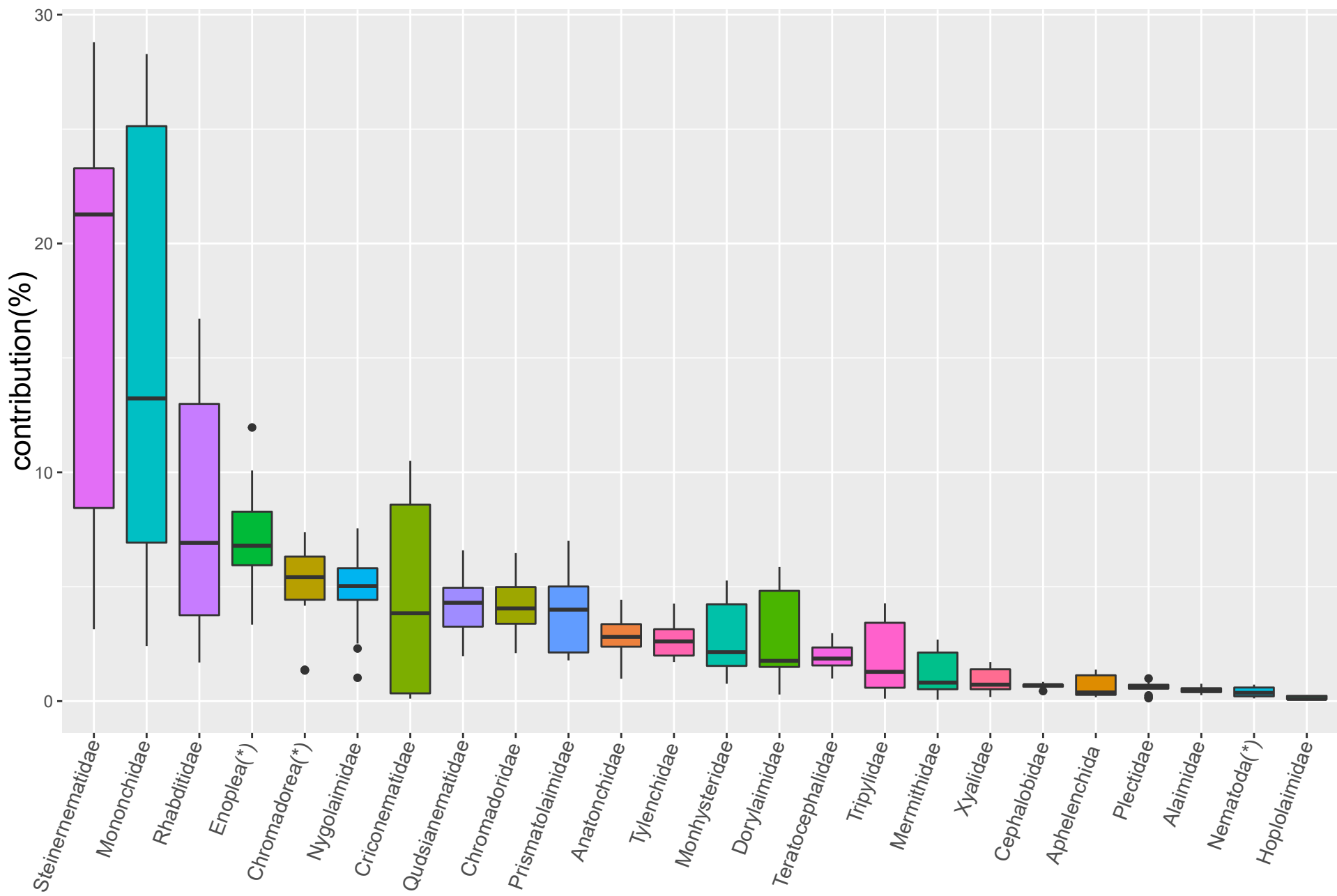

Supplement: Supplementary file 3 — Fig S3 [file ECE3-11-18540-s006.pdf]

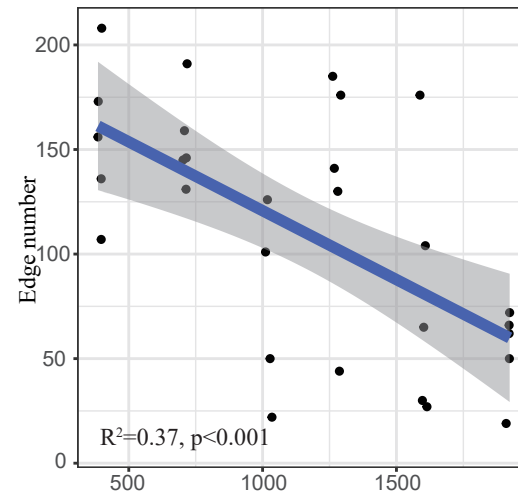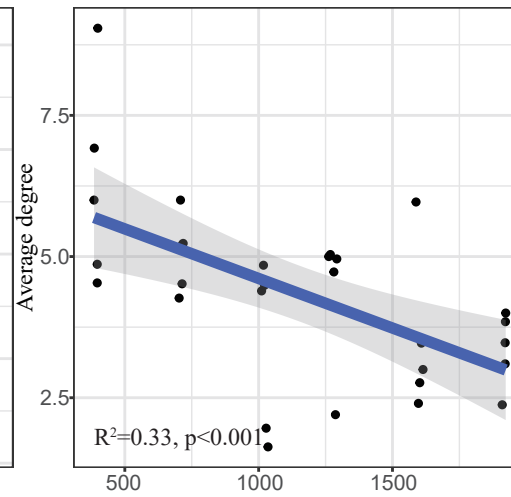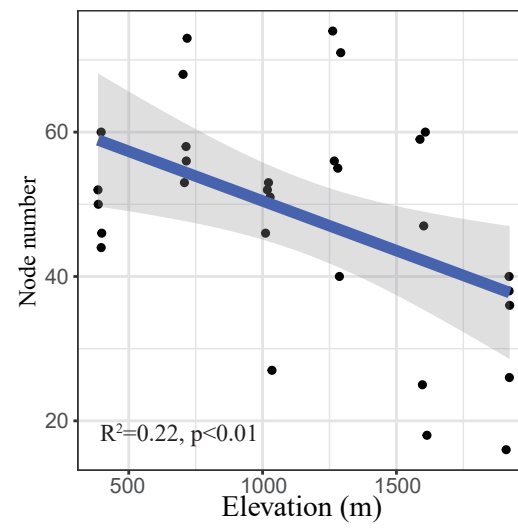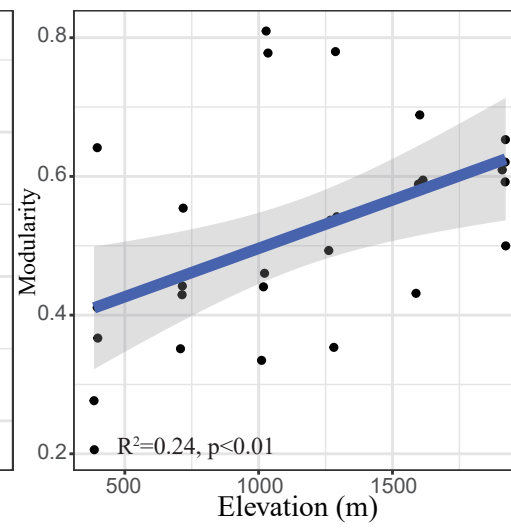

Supplement: Supplementary file 4 — Fig S4 [file ECE3-11-18540-s001.pdf]

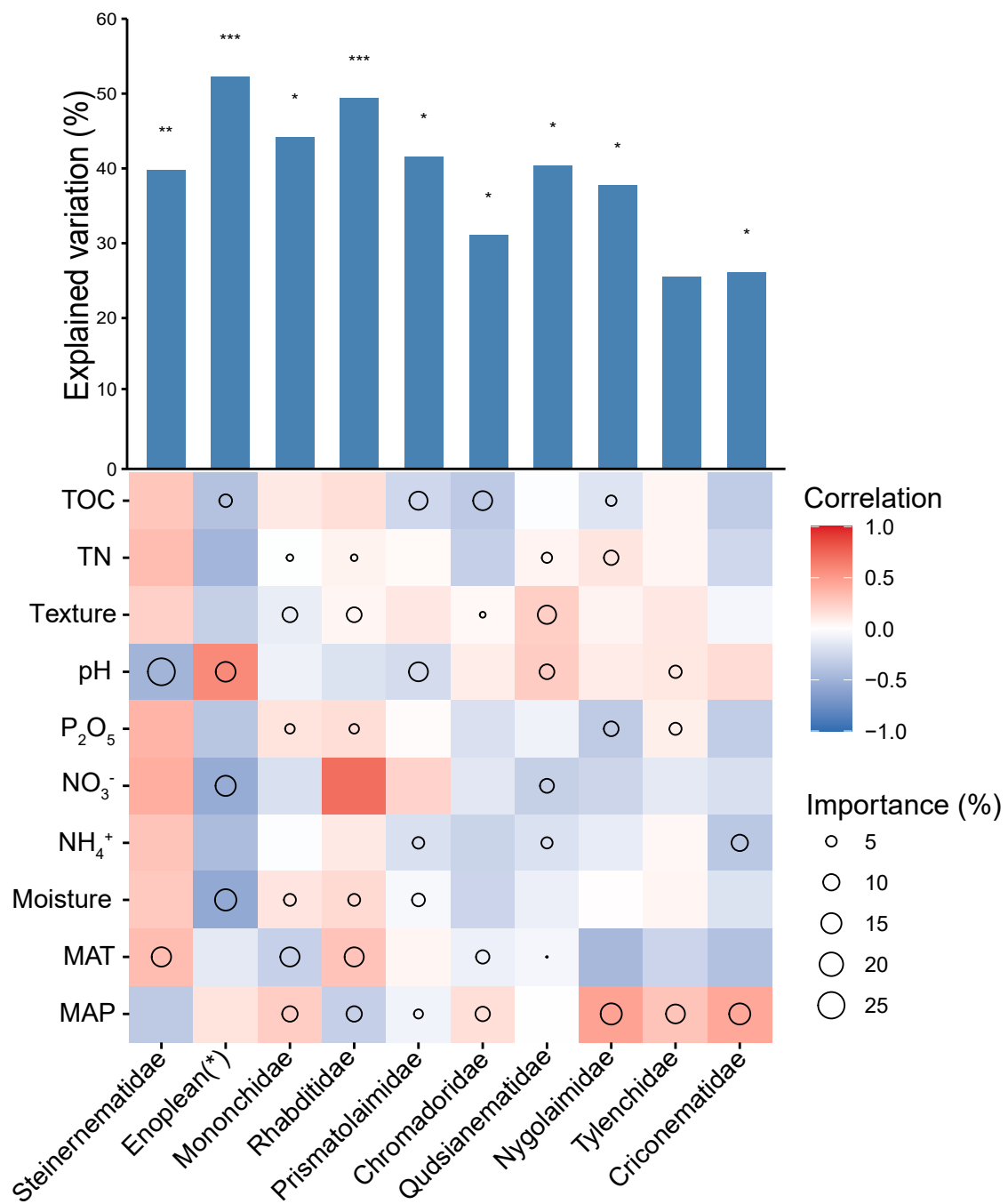

Supplement: Supplementary file 5 — Fig S5 [file ECE3-11-18540-s002.pdf]

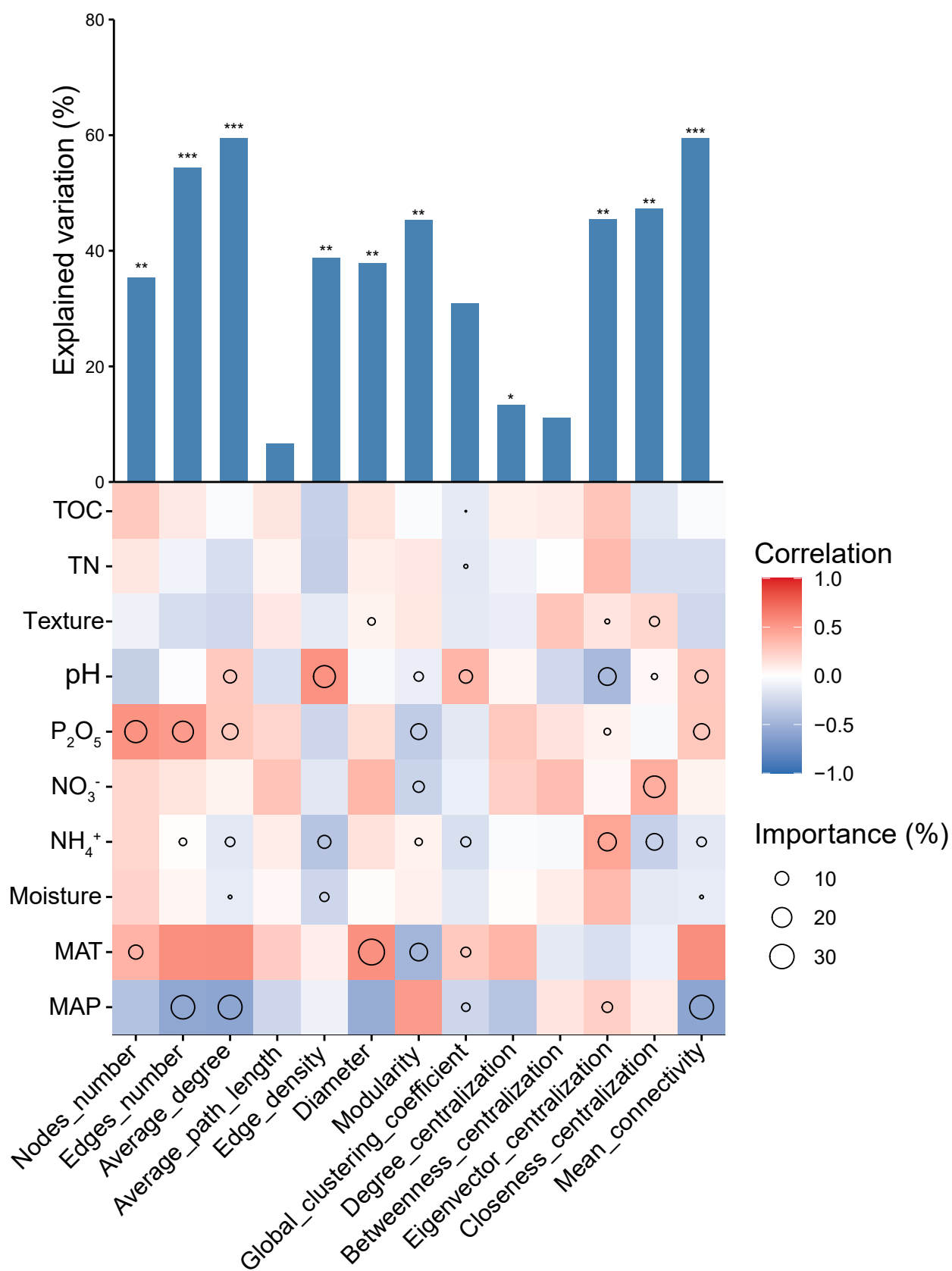

Supplement: Supplementary file 6 — Fig S6 [file ECE3-11-18540-s004.pdf]

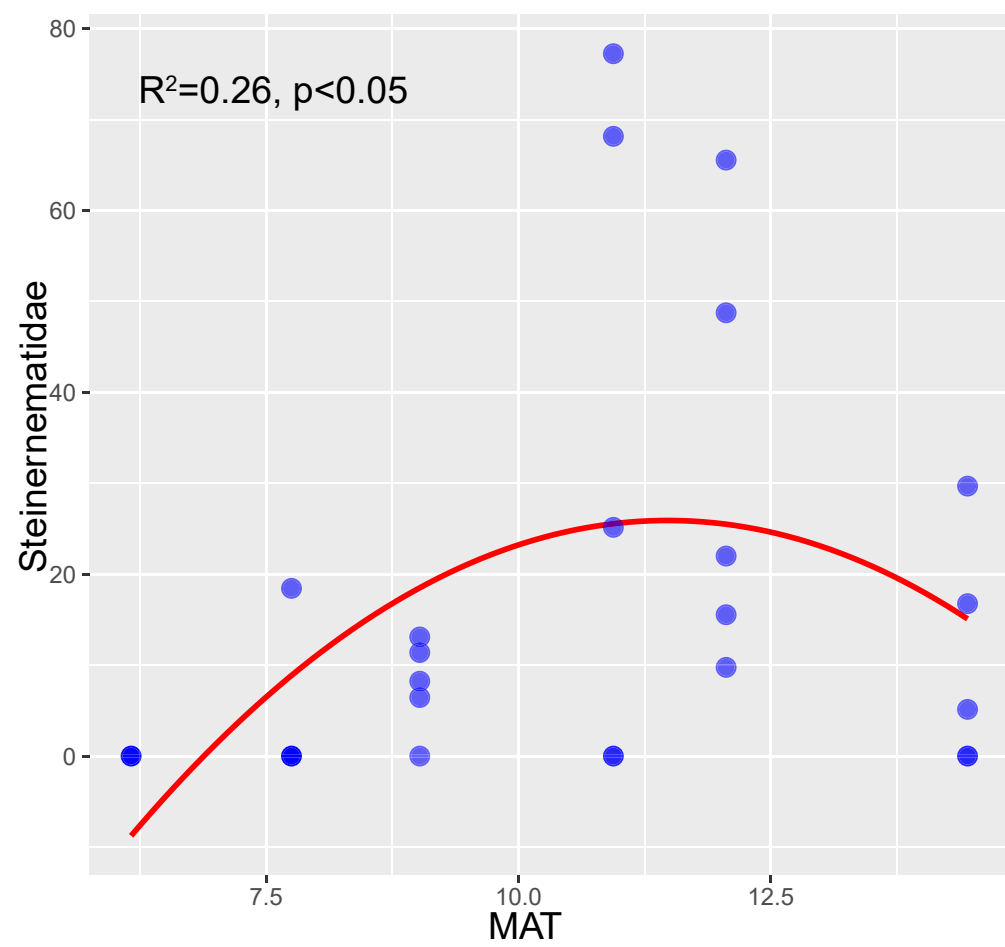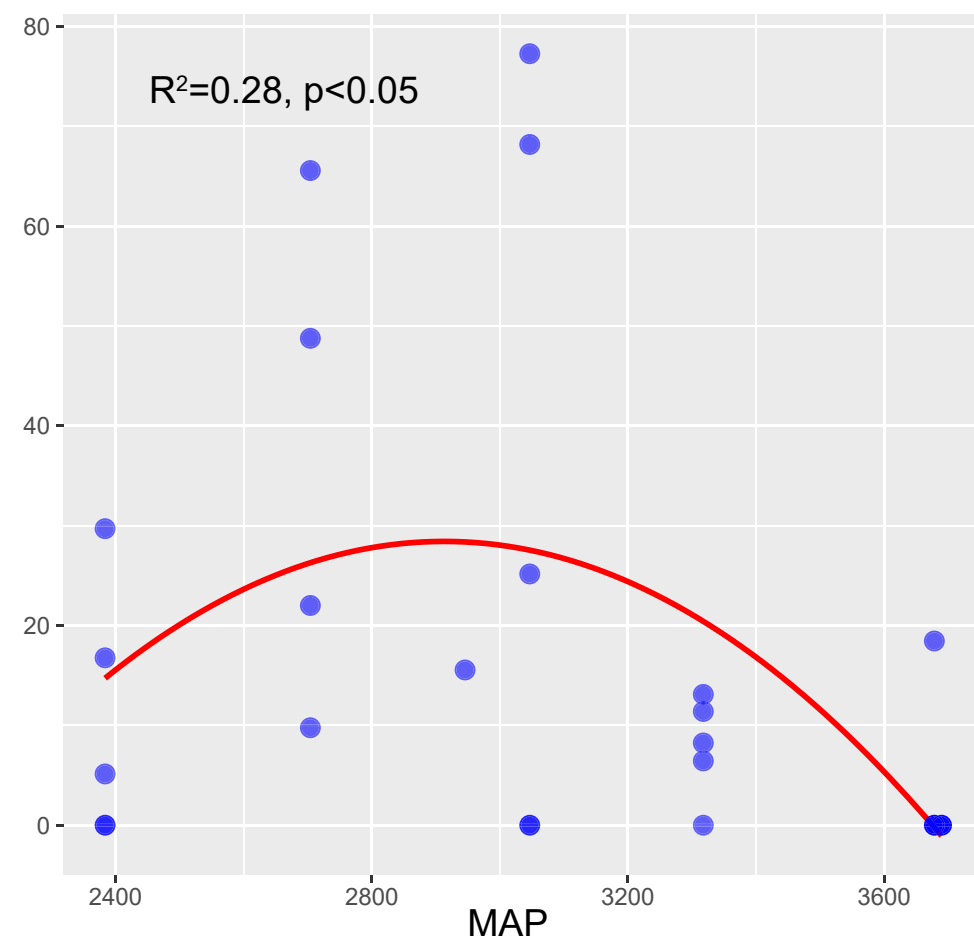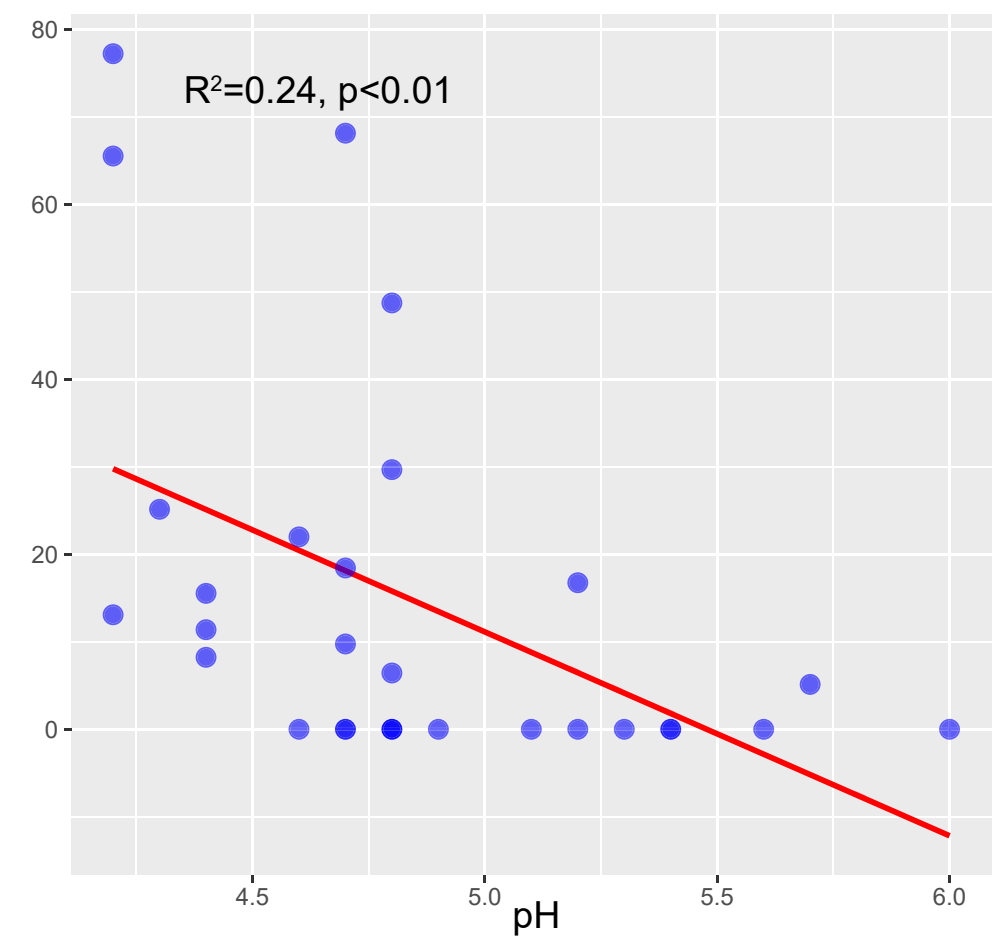

Supplement: Supplementary file 7 — Fig S7 [file ECE3-11-18540-s003.pdf]
