# Supplementary material for: Elevation‐related climatic factors dominate soil free‐living nematode communities and their co‐occurrence patterns on Mt. Halla, South Korea
Source: Ecol Evol. 2021 Dec 15;11(24):18540–51. doi: 10.1002/ece3.8454 (PMC8717350; doi:10.1002/ece3.8454)
Supplement: Supplementary file 8 — Appendix S1 [file ECE3-11-18540-s007.docx]

**Supporting Information**

**Elevation-related climatic factors dominate soil free-living nematode communities and their co-occurrence patterns on Mt. Halla, South Korea**

Supplementary Tables S1, S2, S3, S4 and S5

Supplementary Figures S1, S2, S3, S4, S5, S6 and S7

**Table. S1** Description of node-level and network-level topological features in the co-occurrence network for soil nematode communities on Mt. Halla.

| Level | Topological feature | Description |
| --- | --- | --- |
|  | Degree | The number of connections for a single node. |
|  | Betweenness centrality | In the network, any two nodes have the shortest path, and the betweenness centrality of a node is the number of times the shortest path passes through the node. |
| Node-level | Eigenvector centrality | A relative score measurement of the influence of a single node on the network. A high feature vector score means that a node is connected to many nodes with a high score. |
|  | Closeness centrality | The average of the shortest path length from the node to every other node in the network. |
|  | Node number | Number of nodes |
|  | Edge number | Number of potential links between associated nodes |
|  | Average path length | The average value of the distance between all pairs of nodes in the network |
|  | Average degree | The average number of edges per node in the network |
|  | Edge density | The ratio of the number of edges and the number of possible edges |
| Network-level | Diameter | The shortest distance between the two furthest nodes in the network |
|  | Modularity | Measures the strength of dividing the network into modules |
|  | Global clustering coefficient | The degree to which nodes in the network tend to cluster together |
|  | Degree centralization | Creating a network level centralization measure from the degree centrality scores of the nodes |
|  | Betweenness centralization | The mean of betweenness centrality values for all nodes |
|  | Closeness centralization | The mean of closeness centrality values for all nodes |
|  | Eigenvector centralization | The mean of eigenvector centralization values for all nodes |
|  | Mean connectivity | The average degree of interconnection between the various parts of the network |

**Table. S2** Dissimilarity in nematodes community composition (based on Bray-Curtis distance) between elevations as determined by permutational multivariate analysis of variance (PERMANOVA). ALL Nematodes: whole nematode; BF Nematodes: bacteria-feeding nematodes; OP Nematodes: omnivore/predator nematodes. *P < 0.05, **P < 0.01 and ***P < 0.001.

|  | ALL Nematodes | BF Nematodes | OP Nematodes |
| --- | --- | --- | --- |
|  | R^2^ | R^2^ | R^2^ |
| ALL Site | 0.40*** | 0.38** | 0.39*** |
| H400m vs H700m | 0.36** | 0.34* | 0.36* |
| H400m vs H1000m | 0.25* | 0.28* | 0.19 |
| H400m vs H1300m | 0.36** | 0.23* | 0.40* |
| H400m vs H1600m | 0.27* | 0.15 | 0.31* |
| H400m vs H1900m | 0.27** | 0.37** | 0.31* |
| H700m vs H1000m | 0.18 | 0.38** | 0.09 |
| H700m vs H1300m | 0.38* | 0.46** | 0.37* |
| H700m vs H1600m | 0.39* | 0.14 | 0.39* |
| H700m vs H1900m | 0.39** | 0.55** | 0.42** |
| H1000m vs H1300m | 0.22 | 0.13 | 0.19 |
| H1000m vs H1600m | 0.25* | 0.17 | 0.20 |
| H1000m vs H1900m | 0.27** | 0.32** | 0.23* |
| H1300m vs H1600m | 0.25* | 0.21 | 0.24 |
| H1300m vs H1900m | 0.28* | 0.41* | 0.30* |
| H1600m vs H1900m | 0.17* | 0.20 | 0.13 |

**Table. S3** Soil nematode co-occurrence network topological feature related statistics on Mt. Halla.

| **Index** | | **Empirical network** | |
| --- | --- | --- | --- |
| Node number | 311 | |  |
| Edge number | 930 | |  |
| Average degree | 5.981 | |  |
| Density | 0.019 | |  |
| Diameter | 14 | |  |
| Modularity | 0.720 | |  |
| Average path length | 5.614 | |  |
| Clustering coefficient | 0.521 | |  |
| Degree centralization | 0.049 | |  |
| Eigenvector centralization | 0.924 | |  |

**Table. S4** Mantel tests for the relationship between nematodes community composition and environmental variables. ALL: whole nematode; BF: bacteria-feeding nematodes; OP: omnivore/predator nematodes. The climatic factors include mean annual precipitation (MAP), mean annual temperature (MAT); The edaphic factors include soil pH (pH), total organic carbon (TOC), total nitrogen (TN), ammonium (NH_4_^+^), nitrate (NO_3_^-^), available phosphate (P_2_O_5_), soil moisture (Moisture), and soil texture (Texture). *P < 0.05, **P < 0.01 and ***P < 0.001; †, pH was marginally significant.

|  | Mt. Halla ALL | | Mt. Halla BF | | Mt. Halla OP | |
| --- | --- | --- | --- | --- | --- | --- |
| Name | Mantel |  | Mantel |  | Mantel |  |
| MAP | 0.41*** |  | 0.11 |  | 0.41*** |  |
| MAT | 0.44*** |  | 0.12 |  | 0.44*** |  |
| pH | 0.41** |  | 0.19^†^ |  | 0.32*** |  |
| TOC | 0.12 |  | 0.04 |  | 0.08 |  |
| TN | 0.23** |  | 0.09 |  | 0.22** |  |
| NH_4_^+^ | 0.21** |  | 0.09 |  | 0.21** |  |
| NO_3_^-^ | 0.06 |  | 0.18 |  | -0.02 |  |
| P_2_O_5_ | 0.05 |  | -0.07 |  | 0.04 |  |
| Texture | 0.07 |  | -0.02 |  | -0.01 |  |
| Moisture | 0.21** |  | 0.03 |  | 0.20** |  |

**Table. S5** Mantel and Partial mantel tests for the relationship between nematodes network topological features and environmental variables. The climatic factors include mean annual precipitation (MAP), mean annual temperature (MAT); The edaphic factors include soil pH (pH), total organic carbon (TOC), total nitrogen (TN), ammonium (NH_4_^+^), nitrate (NO_3_^-^), available phosphate (P_2_O_5_), soil moisture (Moisture), and soil texture (Texture). *P < 0.05, **P < 0.01 and ***P < 0.001.

| Name | Mental | Partial mental |
| --- | --- | --- |
| MAP | 0.254** | 0.252** |
| MAT | 0.211** | -0.102 |
| pH | 0.087 | 0.031 |
| TOC | 0.008 | 0.003 |
| TN | 0.034 | 0.016 |
| NH_4_^+^ | 0.062 | 0.062 |
| NO_3_^-^ | -0.102 | -0.097 |
| P2O5 | 0.198* | 0.186* |
| Texture | 0.001 | -0.009 |
| Moisture | -0.009 | -0.029 |


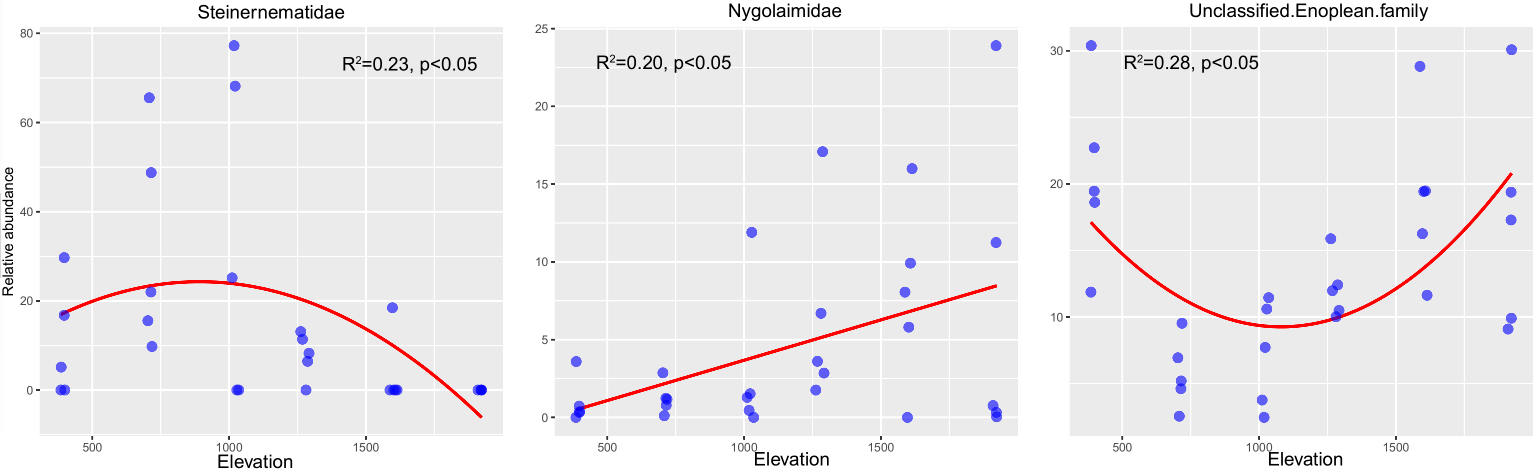


**Fig. S1** The linear regression model of the relative abundance of Steinernematidae, Nygolaimidae, and unclassified Enoplean family along the elevation.


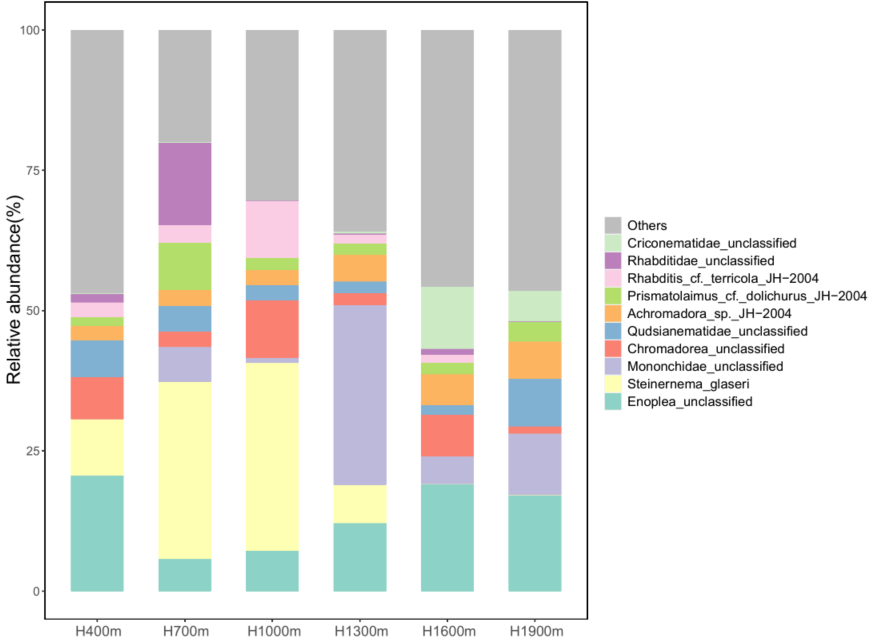


Fig. S2 Relative abundance (%) of nematode genus on different elevational isoclines.


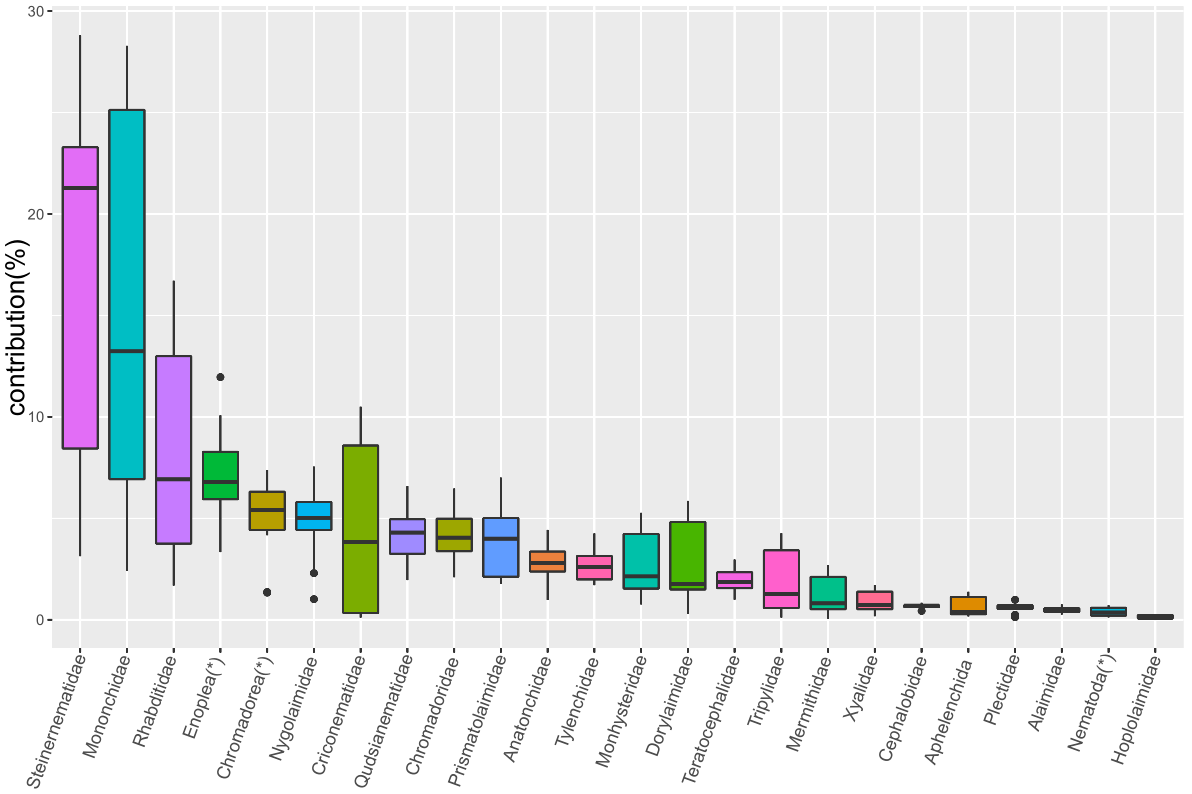


**Fig. S3** The contributions of nematode families which generated the community difference between the elevational transect of soils. Enoplea (*): unclassified Enoplean family; Chromadorea (*): unclassified Chromadorea family; Nematoda (*): unclassified Nematoda family.


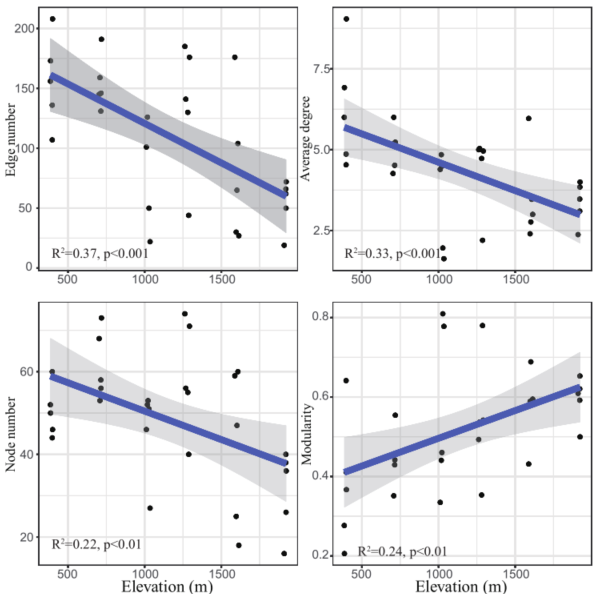


**Fig. S4** Linear regression model of elevation and main network topology features.


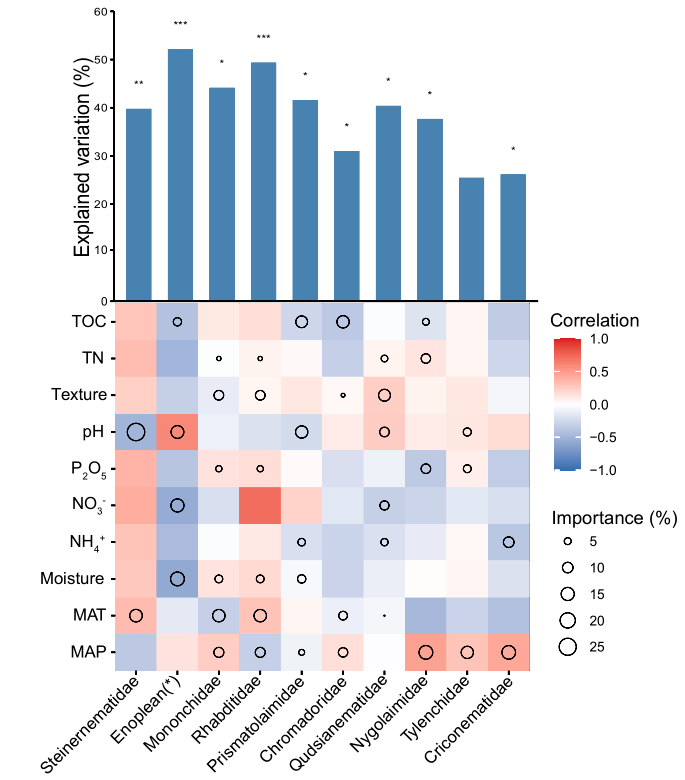


**Fig. S5** The correlation between environmental factors and main nematode families along with the best multiple regression model. Circle size represents the variable’s importance. Colors represent Spearman correlations. The climatic factors include mean annual precipitation (MAP), mean annual temperature (MAT); The edaphic factors include soil pH (pH), total organic carbon (TOC), total nitrogen (TN), ammonium (NH_4_^+^), nitrate (NO_3_^-^), available phosphate (P_2_O_5_), soil moisture (Moisture), and soil texture (Texture). *P < 0.05, **P < 0.01 and ***P < 0.001.


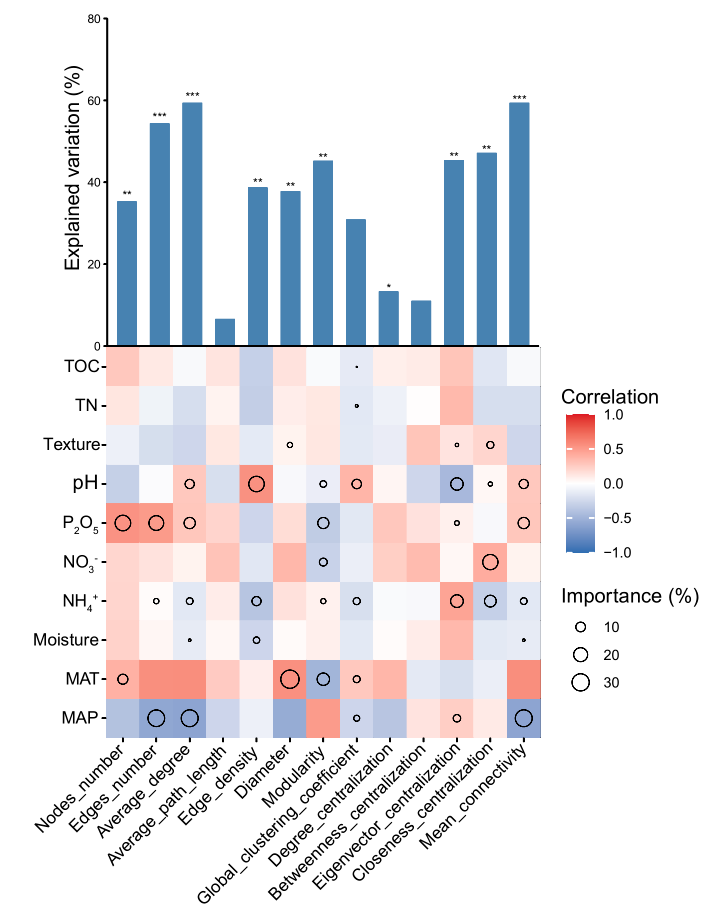


**Fig. S6** The correlation between environmental factors and network topology features and the best multiple regression model. The climatic factors include mean annual precipitation (MAP), mean annual temperature (MAT); The edaphic factors include soil pH (pH), total organic carbon (TOC), total nitrogen (TN), ammonium (NH_4_^+^), nitrate (NO_3_^-^), available phosphate (P_2_O_5_), soil moisture (Moisture), and soil texture (Texture). *P < 0.05, **P < 0.01 and ***P < 0.001.


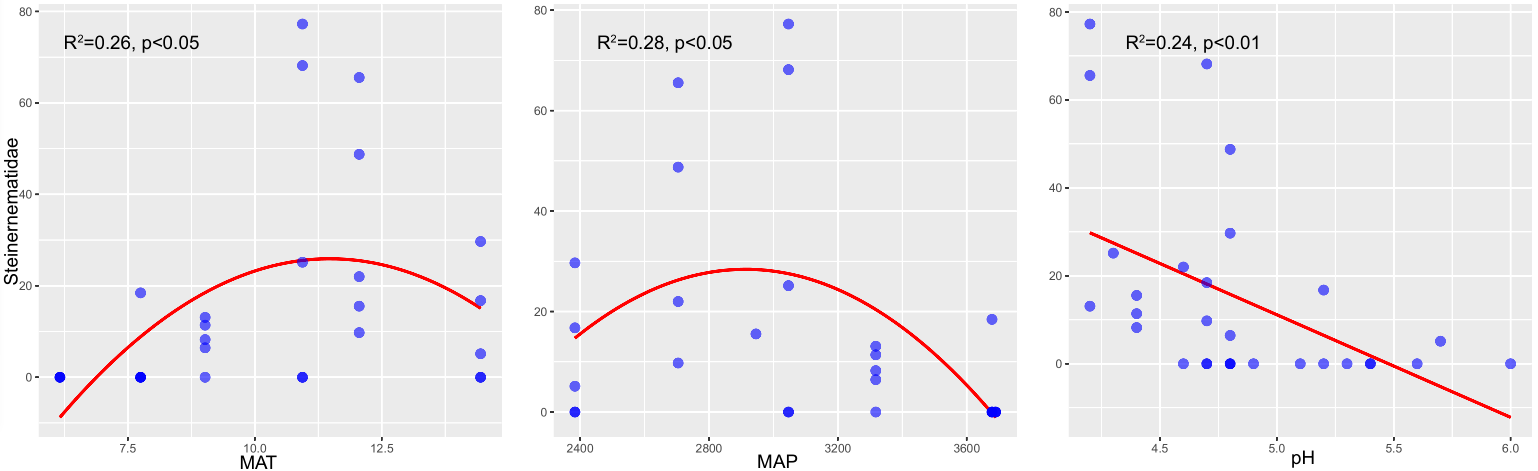


**Fig. S7** The linear regression model of the relative abundance of Steinernematidae with MAT, MAP, and pH. MAT: mean annual temperature; MAP: mean annual precipitation; pH: soil pH.
